# An Ixodes scapularis Protein Disulfide Isomerase Contributes to Borrelia burgdorferi Colonization of the Vector

**DOI:** 10.1128/IAI.00426-20

**Published:** 2020-11-16

**Authors:** Yongguo Cao, Connor Rosen, Gunjan Arora, Akash Gupta, Carmen J. Booth, Kristen E. Murfin, Jiri Cerny, Alejandro Marin Lopez, Yu-Min Chuang, Xiaotian Tang, Utpal Pal, Aaron Ring, Sukanya Narasimhan, Erol Fikrig

**Affiliations:** a Department of Clinical Veterinary Medicine, and Key Laboratory for Zoonosis Research, Ministry of Education, College of Veterinary Medicine, Jilin University, Changchun, China; b Section of Infectious Diseases, Department of Internal Medicine, Yale University School of Medicine, New Haven, Connecticut, USA; c Department of Immunobiology, Yale University School of Medicine, New Haven, Connecticut, USA; d Department of Comparative Medicine, Yale University School of Medicine, New Haven, Connecticut, USA; e Department of Veterinary Medicine, University of Maryland, College Park, Maryland, USA; f Howard Hughes Medical Institute, Chevy Chase, Maryland, USA; Stanford University

**Keywords:** *Borrelia burgdorferi*, *Ixodes scapularis*, colonization, protein disulfide isomerase

## Abstract

Borrelia burgdorferi causes Lyme disease, the most common tick-transmitted illness in North America. When Ixodes scapularis feed on an infected vertebrate host, spirochetes enter the tick gut along with the bloodmeal and colonize the vector. Here, we show that a secreted tick protein, I. scapularis protein disulfide isomerase A3 (IsPDIA3), enhances B. burgdorferi colonization of the tick gut. I. scapularis ticks in which *ispdiA3* has been knocked down using RNA interference have decreased spirochete colonization of the tick gut after engorging on B. burgdorferi-infected mice. Moreover, administration of IsPDIA3 antiserum to B. burgdorferi-infected mice reduced the ability of spirochetes to colonize the tick when feeding on these animals. We show that IsPDIA3 modulates inflammatory responses at the tick bite site, potentially facilitating spirochete survival at the vector-host interface as it exits the vertebrate host to enter the tick gut. These data provide functional insights into the complex interactions between B. burgdorferi and its arthropod vector and suggest additional targets to interfere with the spirochete life cycle.

## Abstract (precis)

Borrelia burgdorferi causes Lyme disease, the most common tick-transmitted illness in North America. When Ixodes scapularis feed on an infected vertebrate host, spirochetes enter the tick gut along with the bloodmeal and colonize the vector. Here, we show that a secreted tick protein, I. scapularis protein disulfide isomerase A3 (IsPDIA3), enhances B. burgdorferi colonization of the tick gut.

## INTRODUCTION

Ixodes scapularis, the black-legged or deer tick, is a vector for multiple human pathogens, including Borrelia (Borreliella) burgdorferi, the agent of Lyme disease. I. scapularis ticks are obligate hematophagous arthropods that feed on mammalian hosts by tearing the host skin with their mouth parts, damaging vessels, and obtaining blood from the ensuing hematoma (1, 2). Tick saliva spit into the feeding lesion contains an array of immunomodulatory molecules. These molecules enable I. scapularis to circumvent and defuse host immune responses and successfully feed to repletion (3–7). During this process, ticks acquire pathogens from an infected host, or if I. scapularis is infected, transmit the pathogens to the vertebrate host. Pathogens traffic through the complex feeding site during entry into and exit from the mammalian host and benefit from immunomodulatory salivary components (8).

A recent study by Murfin et al. (9) showed that tick salivary proteins also facilitate B. burgdorferi chemotaxis to the tick bite site. A high-molecular-weight salivary fraction that facilitated chemotaxis contained several secreted proteins, including, an I. scapularis protein disulfide isomerase A3, here named IsPDIA3. PDIA3 represents an abundant endoplasmic reticulum-associated protein of the thioredoxin family of protein disulfide isomerases. This protein is involved in the processes of oxidation and isomerization of intramolecular disulfide bonds, ensuring proper folding of the nascent polypeptides (10). PDIs are generally dimers of 2 identical subunits and contain two thioredoxin domains as the active sites to play a homeostatic role in redox control at the cell surface (11). This homeostasis is carried out primarily by the redox exchange between PDI (predominantly oxidized) and PDIA3 (predominantly reduced) (10). The PDIA3 protein consists of 4 thioredoxin-like domains; the a and a′ domains have Cys-Gly-His-Cys active site motifs (CGHC motifs) and are catalytically active, while the b and b′ domains contain a calnexin (CNX) binding site, and this binding is critical to ensure proper protein folding in the endoplasmic reticulum (12). PDIA3 is also part of the MHC-I peptide loading complex, which is essential for formation of the final antigen conformation and export from the endoplasmic reticulum to the cell surface (13–15). In humans, PDIA3 (also known as ERp57, ERp60, GRP58, and 1,25D3-MARRS), in contrast to the 20 other PDI family members, is present not only in the endoplasmic reticulum but also in the nucleus, extracellular matrix, and plasma membrane (16), suggesting that the enzymatic activity of PDIA3 likely modulates diverse biological functions (13–15, 17).

Murfin et al. (9) and Kim et al. (18) showed that IsPDIA3 is present in tick saliva and is secreted into the host, suggesting that in I. scapularis IsPDIA3 is a secreted protein and may perform additional functions at the tick-host interface that impact pathogen transmission or colonization. While PDI homologs have been described from ticks (19, 20), their physiological functions are not well understood (21). In this study, we examine the biological role of IsPDIA3 in the context of B. burgdorferi life cycle and demonstrate that IsPDIA3 facilitates spirochete colonization of the tick gut. We speculate that the reductase/isomerase function of IsPDIA3 modulates this process. Whether this function is critical at the tick-host interface or in the gut or both remains to be elucidated.

## RESULTS

### IsPDIA3 is expressed in I. scapularis and secreted in saliva.

**FIG 1:**
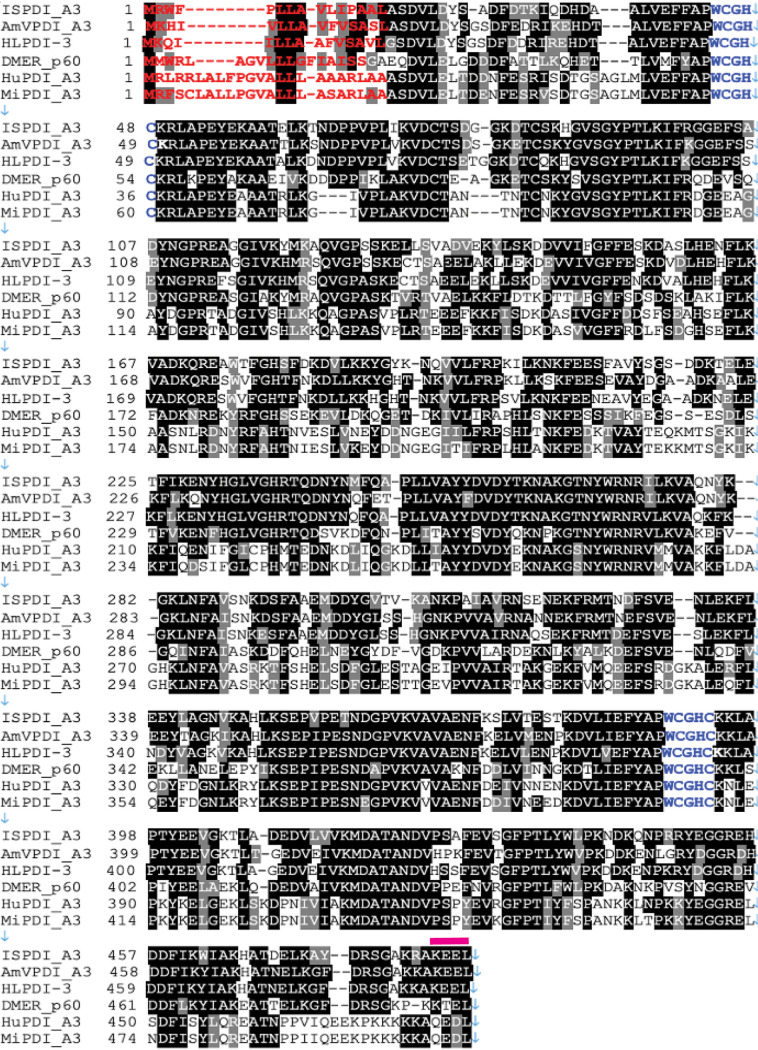
Protein sequence comparison of IsPDIA3. Multiple-sequence alignment of Ixodes scapularis *IsPDI* aligned with nine homologs of protein disulfide isomerase (PDI) in Amblyomma variegatum (AmVPDI_A3), Haemaphysalis longicornis (HLPDI_A3), Drosophila melanogaster (DMPDI_p60), Homo sapiens (HuPDI_A3), and Mus musculus (MiPDI_A3). Amino acids shown in black are identical. Amino acids shown in red denote the predicted signal peptide sequence, blue letters label the thioredoxin motif, and pink bars above the sequence indicate the endoplasmic reticulum retention signal.

**FIG 2:**
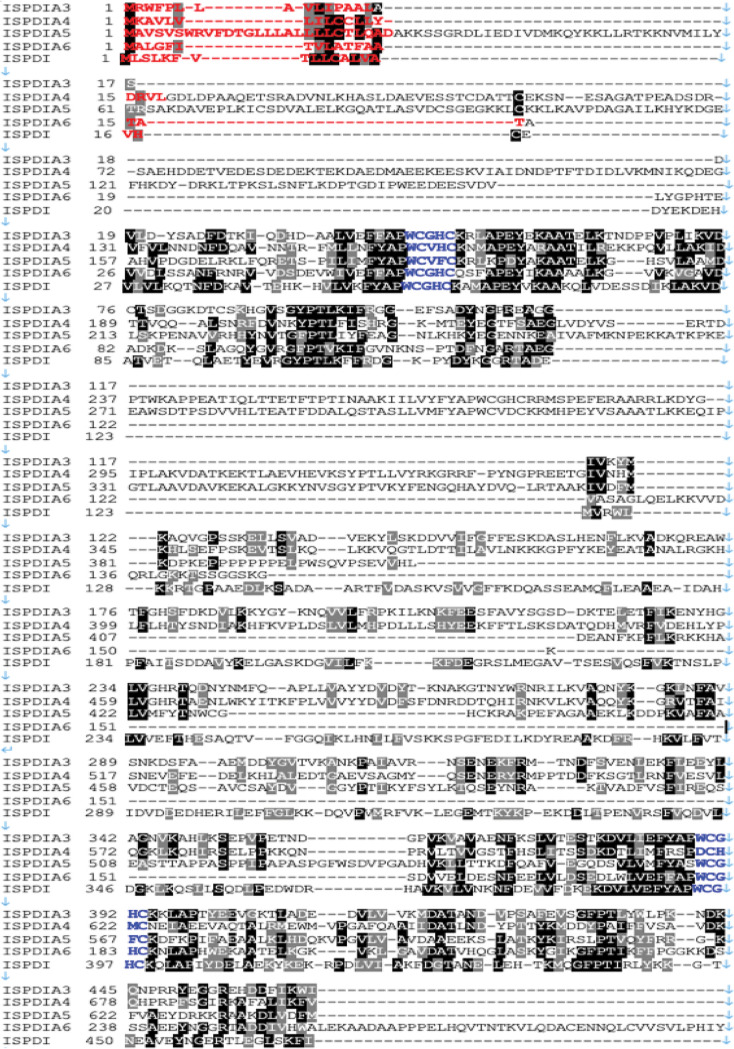
Protein sequence comparison of IsPDIA3. Multiple-sequence alignment of I. scapularis *IsPDI* aligned with 4 paralogs of protein disulfide isomerase (PDI) in I. scapularis. Amino acids shown in black are identical. Amino acids shown in red denote the predicted signal peptide sequence, blue letters label the thioredoxin motif, and pink bars above the sequence indicate the endoplasmic reticulum retention signal.

ISCW016161 (www.vectorbase.org), gene name for a protein that encodes a putative I. scapularis protein disulfide isomerase A3, here referred to as IsPDIA3, and the full-length open reading frame was cloned from nymphal salivary gland cDNA. Protein sequence alignment of IsPDIA3 with protein disulfide isomerase (PDI) homologs from other species showed that the protein is highly conserved across the animal kingdom (Fig. 1) and that all PDIs contain two canonical thioredoxin motifs (Fig. 1). IsPDIA3 shares 60 to 70% homology with other tick and Drosophila orthologs and 20 to 50% homology with human and mouse orthologs. IsPDIA3 contains a signal peptide (Fig. 1), suggesting that it is likely to be a secreted protein despite encoding an endoplasmic retention signal in its carboxy terminus. PDIs in other arthropods, mice, and humans also contain a signal peptide (Fig. 1). In addition to IsPDIA3, I. scapularis has four additional paralogs with 31 to 48% homology to protein disulfide isomerases, including IsPDI (locus: www.vectorbase.org; LOC8037869), IsPDIA4 (LOC8024123), IsPDIA5 (LOC8038380), and IsPDIA6 (LOC8027597) (Fig. 2), and all four contain a signal peptide, suggesting that these may also be secreted proteins despite containing an endoplasmic retention signal in their carboxy terminus (Fig. 2).

**FIG 3:**
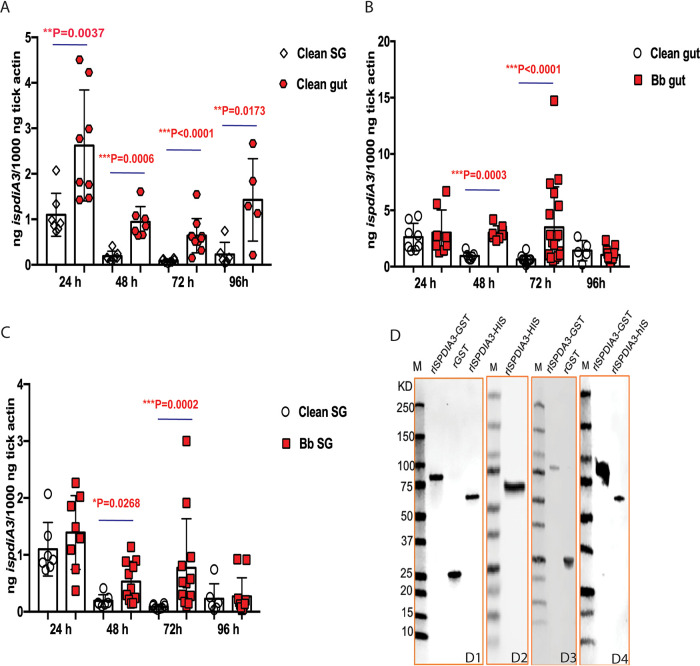
Expression profile of IsPDIA3. (A) IsPDIA3 transcript expression profile in guts (gut) and salivary glands (SG) in pathogen-free (clean) nymphs after 24, 48, 72, and 96 h of feeding. Each data point represents a pool of three nymphal salivary glands or guts. (B) Gut and (C) SG of clean nymphs and *Borrelia*-infected (Bb) nymphs after 24, 48, 72, and 96 h of feeding. Each data point represents a pool of three nymphal salivary glands or guts. (D) Coomassie stain of rIsPDIA3-GST, rGST, rIsPDIA3-HIS (D1); Western blot assessment of: rIsPDIA3-His using anti-His antibody (D2), rIsPDIA3-GST using anti-GST antibody (D3); and rIsPDIA3-GST and rIsPDIA3-HIS using anti-rIsPDIA3-GST rabbit antiserum (D4). Results represent mean ± standard deviation (SD) of values.

*ispdiA*3 mRNA transcripts were detected in both salivary glands and guts of clean nymphs throughout the course of tick feeding, and expression in the tick guts was significantly higher at all time points (*P* < 0.05) compared to that in tick salivary glands (Fig. 3A). To determine whether the expression of *ispdiA3* was altered by the presence of B. burgdorferi, we assessed *ispdiA3* expression in infected and uninfected nymphs. These data showed that *ispdiA3* expression was significantly increased in B. burgdorferi-infected tick guts (Fig. 3B) and in salivary glands at 48 and 72 h of tick feeding (Fig. 3C) compared to that in uninfected guts and salivary glands.

To evaluate the importance of IsPDIA3 in the context of tick-pathogen interactions, we generated recombinant IsPDIA3 as a glutathione *S*-transferase (GST)-tagged protein (rIsPDIA3-GST) in a prokaryotic expression system using the pGEX vector and in a eukaryotic expression system as a histidine (His)-tagged protein (rIsPDIA3-His) using the mammalian expression vector pEZT-D-lux plasmid. Recombinant proteins were purified to homogeneity (Fig. 3D, subpanel D1) as described in Materials and Methods. Polyclonal antibodies against each of the rIsPDIA3 proteins were raised in rabbits, and seroreactivity to the recombinant proteins was confirmed by Western blot (Fig. 3D, subpanels D2 to D4).

### RNAi-mediated knockdown of IsPDIA3 impairs B. burgdorferi colonization.

**FIG 4:**
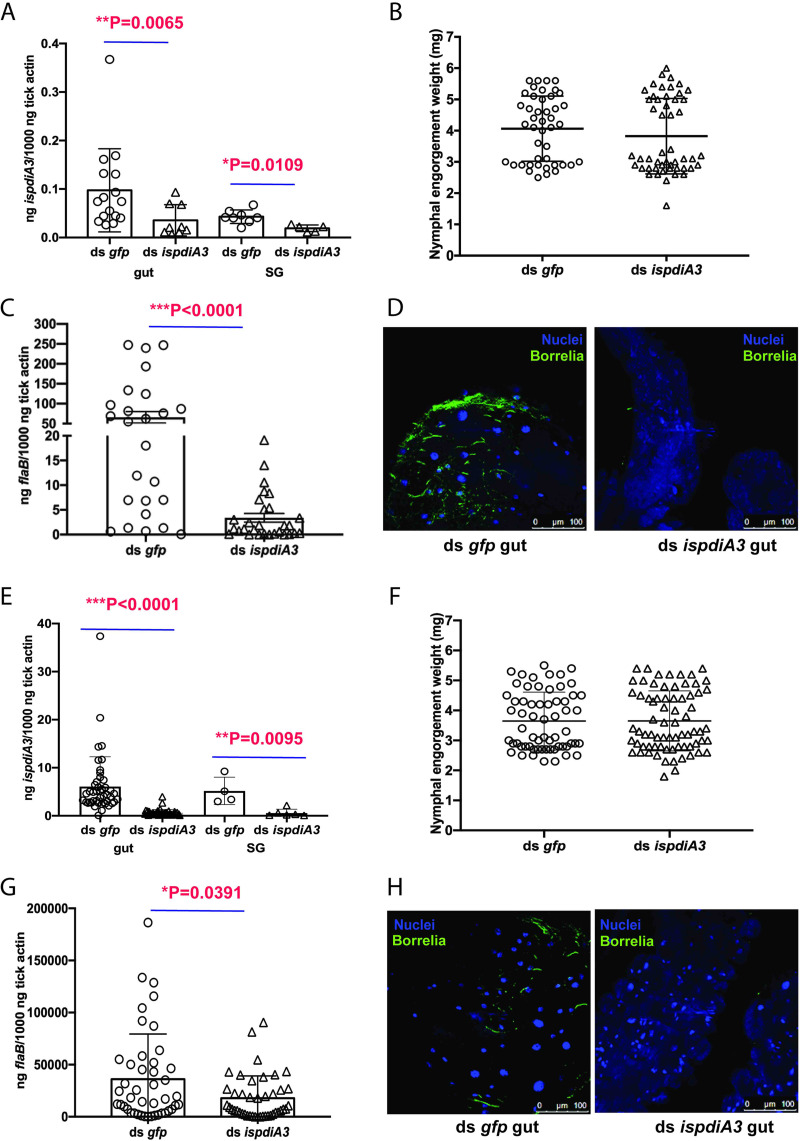
Knockdown of *ispdiA3* expression decreases B. burgdorferi colonization by I. scapularis ticks. mRNA transcription was knocked down in I. scapularis salivary glands or gut by body microinjection (A to D) or by anal pore injection (E to H) of ds *ispdia3*. Knockdown efficiency was measured in I. scapularis nymph salivary glands (SG) and gut (gut). Each data point represents a pool of three nymphal salivary glands or guts (A and E). The impact of *ispdia3* knockdown on tick feeding was measured by engorgement weights. Each data point represents a replete nymph (B and F). B. burgdorferi burden in replete tick guts (C and G) was determined by qRT-PCR of *flaB* transcripts, and data were normalized to tick *actin* and visualized by immunofluorescence microscopy using rabbit polyclonal B. burgdorferi antibodies. Each data point represents a pool of three nymphal guts (D and H). Nuclei stained with 4′,6-diamidino-2-phenylindole (DAPI). Magnification is ×40. Data in panels A to H represent averages of 3 biological replicates. Results represent mean ± SD of values. Statistical significance was assessed using a nonparametric Mann-Whitney test; *, *P* < 0.05; **, *P* < 0.01; ***, *P* < 0.001.

To assess whether IsPDIA3 is involved in B. burgdorferi colonization, we performed RNAi-mediated knockdown experiments with I. scapularis nymphs. Double-stranded *ispdiA3* RNA (ds *ispdiA3*) was injected into the idiosoma of uninfected nymphal ticks to knock down expression in the salivary glands. The survival rate of ticks after injection is more than 80%. ds *ispdiA3*-injected nymphs were fed to repletion on B. burgdorferi-infected mice. Control ticks were injected with ds green florescent protein RNA (ds *gfp*) to serve as an irrelevant double-stranded RNA (dsRNA) control. RNA interference (RNAi)-mediated knockdown of *ispdiA3* resulted in an approximately 50% decrease (*P* < 0.05) in transcript levels in salivary glands and in guts at 96 h of feeding (Fig. 4A) compared to that in control ticks injected with ds *gfp*. There were no significant differences in tick feeding as measured by engorgement weights (Fig. 4B). Viable B. burgdorferi burden in guts of replete ticks, as seen by reverse transcription-quantitative PCR (qRT-PCR) assessment of *flaB* transcripts, was significantly lower (*P* < 0.0001) in ds *ispdiA3*-injected ticks compared to that in ds *gfp*-injected ticks (Fig. 4C). Consistent with the quantitative PCR (qPCR) assessment, visualization of B. burgdorferi burden by immunofluorescence microscopy using polyclonal rabbit anti-B. burgdorferi antibodies (Fig. 4D) also showed decreased spirochete burden in ds *ispdi*A3-injected ticks compared to that in ds *gfp*-injected ticks.

In an effort to discern the preferential role of gut or salivary gland (SG)-specific IsPDIA3 on B. burgdorferi colonization, we tried to selectively silence the gut IsPDIA3 by microinjecting ds *ispdiA3* via the anal pore of the tick as described earlier (22). Control ticks were injected with ds *gfp*. RNAi-mediated knockdown of *ispdiA3* resulted in an approximately 90% decrease (*P* < 0.05) of transcript levels in guts at 96 h of feeding (Fig. 4E) compared to that in control ticks injected with ds *gfp*. However, *ispdiA3* transcripts were also significantly reduced in the salivary glands (Fig. 4E). There were no differences in tick feeding as measured by engorgement weights (Fig. 4F). B. burgdorferi burden in guts of replete ticks as seen by qRT-PCR assessment of *flaB* transcripts was significantly decreased (*P* < 0.05) in ds *ispdiA3*-injected ticks compared to that in ds *gfp*-injected ticks (Fig. 4G). Consistent with the qRT-PCR evaluation, visualization of B. burgdorferi burden by immunofluorescence microscopy using polyclonal rabbit anti-B. burgdorferi antibodies (Fig. 4H) also showed decreased spirochete burden in ds *ispdi*A3-injected ticks compared to that in ds *gfp*-injected ticks.

### Humoral responses against IsPDIA3 impair B. burgdorferi colonization of tick gut.

**FIG 5:**
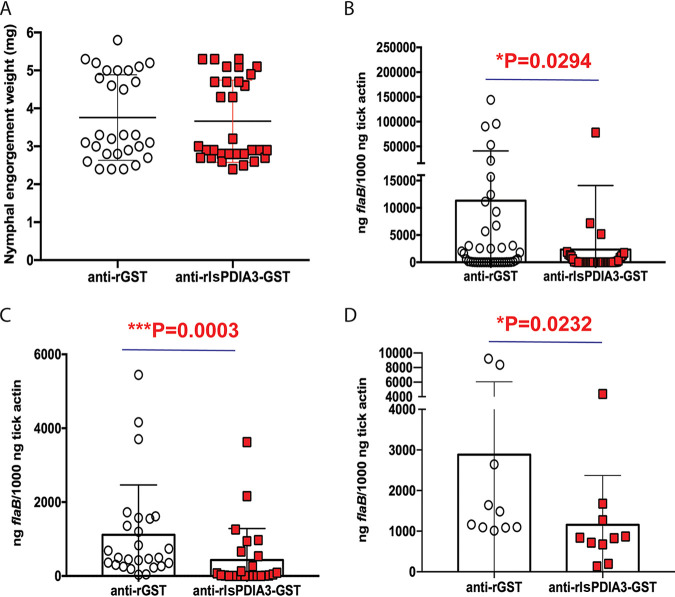
Passive immunization against *IsPDIA3* expression decreases B. burgdorferi colonization by I. scapularis ticks. I. scapularis nymphs were fed on mice passively immunized with polyclonal rabbit rIsPDIA3-GST antiserum or polyclonal rabbit anti-rGST rabbit serum and the impact on (A) feeding as measured by engorgement weights, with each data point representing a replete nymph; (B) B. burgdorferi burden in replete tick guts as measured by qRT-PCR, with each data point representing a pool of two nymphal guts. (C) *Borrelia* burden in pooled larvae fed on mice passively immunized with rIsPDIA3-GST rabbit antiserum or rGST rabbit antiserum, with each data point representing a pool of five larvae; and (D) qRT-PCR assessment of B. burgdorferi burden in nymphs molted from larvae fed on mice passively immunized with rIsPDIA3-GST rabbit antiserum or rGST rabbit antiserum, with each data point representing a pool of three nymphs. Data (A to D) are averages of 3 biological replicates. Results represent mean ± SD of values. Statistical significance was tested using a nonparametric Mann-Whitney test; *, *P* < 0.05; **, *P* < 0.01; ***, *P* < 0.001.

We then wanted to determine whether humoral immunity elicited against IsPDIA3 might impact B. burgdorferi colonization of I. scapularis ticks. Toward this goal, we passively immunized B. burgdorferi-infected mice with rIsPDIA3-GST antiserum and then allowed uninfected I. scapularis nymphs to feed to repletion on these mice. rIsPDIA3-GST rabbit antiserum did not impact nymphal feeding as measured by the comparable engorgement weights of nymphs on rIsPDIA3-GST antiserum-immunized and control rGST antiserum-immunized mice (Fig. 5A). However, B. burgdorferi burden as seen by qRT-PCR assessment of *flaB* transcripts was significantly decreased in the guts of nymphs fed on rIsPDIA3-antiserum-immunized mice compared to that in nymphs fed on control animals (Fig. 5B). This phenotype was also replicated in experiments in which larval ticks were fed on B. burgdorferi-infected mice that were passively immunized with rIsPDIA3 or rGST rabbit antiserum. *Borrelia* burden in larvae fed on mice administered rIsPDIA3 antiserum was significantly decreased compared to that in larvae fed on control animals (Fig. 5C). While molting efficiency of the larvae was not impaired (data not shown), the B. burgdorferi burden in nymphs that had molted from larvae fed on rIsPDIA3 antiserum-immunized mice was significantly decreased compared to that in nymphs molted from larvae fed on control mice (Fig. 5D).

### IsPDIA3 does not influence B. burgdorferi transmission from I. scapularis to the murine host.

**FIG 6:**
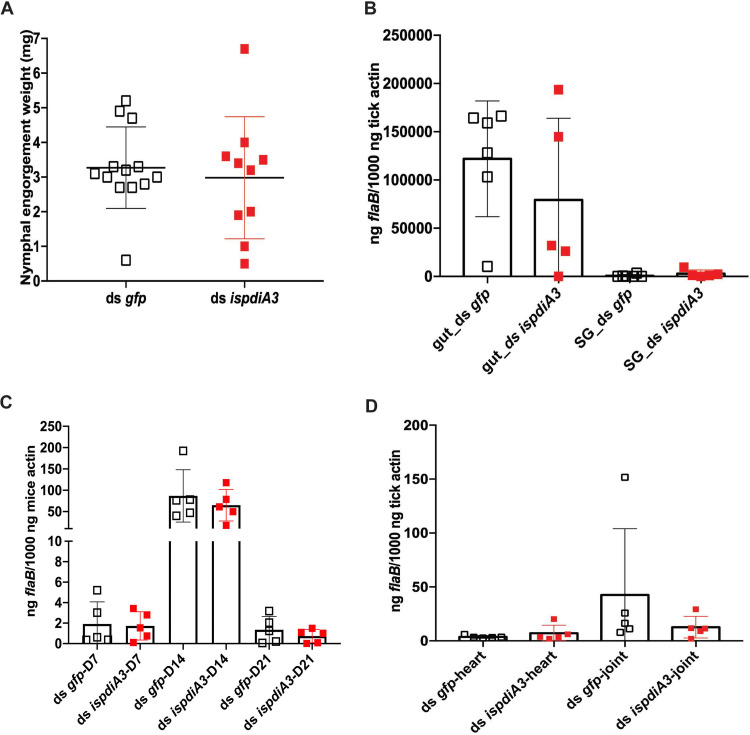
Knockdown of *ispdia3* expression does not impact B. burgdorferi transmission by I. scapularis ticks. *Borrelia*-infected (Bb) nymphs microinjected by body with ds *ispdia3* or ds *gfp* were fed on clean mice to assess transmission of the spirochete. (A) Impact of knockdown of *ispdia3* on engorgement weights; each data point represents a replete nymph. (B) B. burgdorferi burden in salivary glands and guts of replete nymphs, with each data point representing a pool of three nymphal salivary glands and guts, and in (C) mouse skin at 7,14 and 21 days, and (D) and in heart and joint tissues at 21 days was determined by qPCR of *flaB* and normalized to tick or mouse β-*actin*. Horizontal bars represent the mean ± SD. Data in panels A to D are averages of 3 biological replicates. Statistical significance was assessed using a nonparametric Mann-Whitney test (*, *P* < 0.05; **, *P* < 0.01; ***, *P* < 0.001).

To determine whether *IsPDIA3* might also have a role in the transmission of B. burgdorferi to the mammalian host, we performed studies with B. burgdorferi-infected ticks in which *ispdiA3* was silenced using RNAi. Knockdown of *ispdiA3* in B. burgdorferi-infected nymphs by microinjection of ds *ispdiA3* into the idiosoma of ticks did not influence feeding, as seen by comparable engorgement weights in ds *ispdiA3*-injected or ds *gfp*-injected ticks (Fig. 6A). The B. burgdorferi burden, as seen by qRT-PCR assessment of *flaB* transcripts in engorged nymphal tick guts and salivary glands, was also comparable in ds *ispdiA3*- and ds *gfp*-injected ticks (Fig. 6B). B. burgdorferi burdens in the skin of mice (ear skin distal from the tick bite site) at 7, 14, and 21 days post tick detachment (Fig. 6C) and in heart and joint tissues 21 days post tick detachment (Fig. 6D) were also comparable in mice fed upon by ds *gfp*- or by ds *ispdia3*-injected nymphs.

### IsPDIA3 modulates inflammation at the tick bite site.

**FIG 7:**
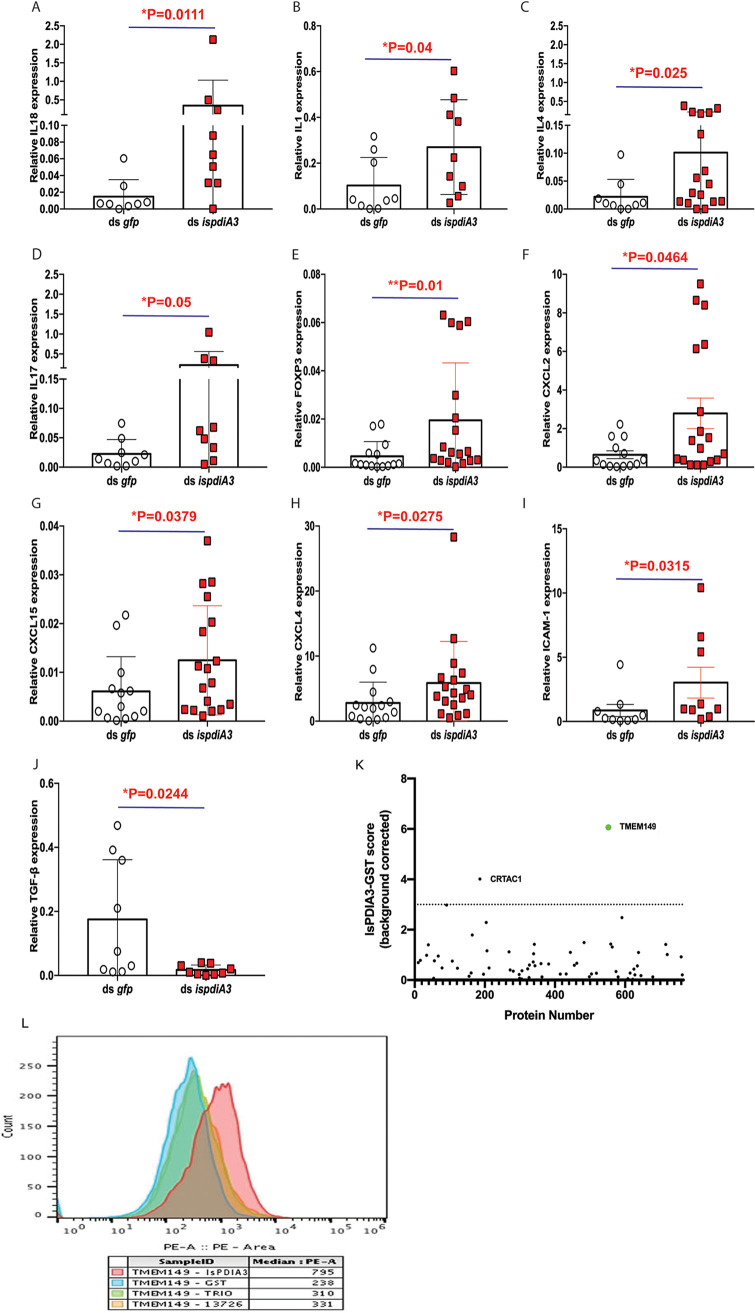
RNAi-mediated knockdown of IsPDIA3 results in an increased inflammatory response at the tick bite site. Clean nymphs microinjected with ds *ispdia3* or ds *gfp* were fed on clean mice to assess expression profiles of cytokines and chemokines by qRT-PCR at 72 h post tick feeding. Expression profiles of (A) interleukin 18 (IL-18), (B) IL-1β, (C) IL-4, (D) IL-17, (E) FOXP3, (F) CXCL2, (G) CXCL15, (H) CXCL4, (I) ICAM-1, and (J) TGF-β. All of these cytokine and chemokine levels in skin tissues were normalized to mouse β-actin RNA levels according to threshold cycle (2^−Δ^*^CT^*) calculations. Data are averages of 3 biological replicates. Results represent mean ± SD of values. Statistical significance was assessed using a nonparametric Mann-Whitney test (*, *P* < 0.05; **, *P* < 0.01; ***, *P* < 0.001). (K) Screening for rIsPDIA3 interactants by yeast display and fluorescence-activated cell sorting (FACS) data show 2 proteins. TMEM149 and CRTAC1, that enriched with rIsPDIA3-GST. (L) FACS assessment of TMEM149 binding to rIsPDIA3-GST, rGST, and GST-tagged Anopheles gambiae salivary proteins TRIO (AGAP001374) or 13726 (AGAP013726).

Since RNAi-mediated silencing of *ispdiA3* in B. burgdorferi-infected ticks did not impact B. burgdorferi burden in the tick during tick-to-mouse transmission, we speculated that IsPDIA3 may function at the tick-host interface to modulate immune responses in favor of B. burgdorferi migration from the host to the tick. Therefore, we sought to determine if immune responses at the tick bite site were altered by IsPDIA3. Towards this understanding, clean nymphs microinjected with ds *ispdia3* or ds *gfp* were fed on the pinnae of clean mice. At 72 h post tick attachment, mice were sacrificed, and ears were dissected for RNA extraction and qRT-PCR assessment of the expression profiles different cytokines and chemokines as described in Materials and Methods. Of the 30 genes (see Table S1 in the supplemental material) assessed, the expression of several genes, including those encoding interleukin 18 (IL-18), IL-1β, IL-4, IL-17, FOXP3, CXCL2, CXCL15, CXCL4, and ICAM-1 (Fig. 7A to I), was significantly increased, and the expression of transforming growth factor β (TGF- β) (Fig. 7J) was significantly decreased at *ispdiA3* knockdown tick bite sites compared to that at control tick bite sites.

### IsPDIA3 interacts with mammalian transmembrane protein 149 or insulin growth factor-like family receptor 1.

To understand how IsPDIA3 modulates inflammatory responses at the tick bite site, we utilized a human exoproteome yeast surface display library as described in Materials and Methods to perform a functional screen for human-secreted or surface-exposed proteins that may interact with rIsPDIA3-GST. Magnetic sorting and enrichment of rIsPDIA3-GST binding clones identified transmembrane protein 149 or insulin growth factor-like family receptor 1 (TMEM149/IGFLR-1) as the strongest interactant compared to other human exoproteins, including chondrocyte-secreted cartilage acidic protein 1 (CRTAC1), the only other exoprotein that showed binding above background (Fig. 7K). rIsPDIA3-GST showed greater than 3-fold binding to TMEM149 compared to rGST or GST-tagged recombinant Anopheles gambiae salivary proteins (AGAP001374; www.vectorbase.org) (Fig. 7L).

## DISCUSSION

Tick salivary and gut proteins have been shown to facilitate pathogen colonization and transmission (8, 23). Proteome analysis of tick saliva and salivary glands (9, 18, 24, 25) has identified numerous proteins in tick saliva, but the functions of these components in the context of tick feeding and pathogen transmission remain to be fully understood. In this study, we examined the functional significance of IsPDIA3, a secreted protein disulfide isomerase (9, 18), on tick feeding, B. burgdorferi colonization of the tick, and spirochete transmission to the murine host.

The predominant function of protein disulfide isomerases is in chaperoning protein folding, thereby ensuring the functional integrity of proteins as they traffic through the endoplasmic reticulum (10). Interestingly, IsPDIA3 has a signal peptide and is shown to be secreted in saliva that is deposited at the bite site in the feeding lesion (9, 18), suggesting potential functions beyond just protein folding. Consistent with this hypothesis, in parasites such as Schistosoma mansoni and Leishmania major, secreted PDIs have been suggested to facilitate parasite-host interactions conducive to successful infection of the host (26). A recent study by Green et al. (27) has shown that a membrane-bound PDI on mammalian neutrophils binds Anaplasma phagocytophilum surface protein Asp14 and facilitates A. phagocytophilum infection of neutrophils. The study also showed that the reductase activity of PDI was critical to facilitate this infection (27) and highlights the potential impact of PDIs in vector-host-pathogen interactions.

When *ispdiA3* expression was knocked down in pathogen-free nymphal ticks by RNAi, B. burgdorferi colonization of the tick gut was significantly diminished. Larvae that fed on mice administered anti-rIsPDIA3 serum also demonstrated decreased B. burgdorferi colonization compared to larvae that fed on mice administered anti-rGST serum. This phenotype was also observed in nymphs that molted from these larvae. This is likely due to decreased spirochete colonization in the larval stage and not due to persistence of anti-IsPDIA3 antibodies during molting. When *ispdia3* expression was knocked down by RNAi in B. burgdorferi-infected ticks, B. burgdorferi burden was not altered in the tick gut or in the salivary glands, suggesting that IsPDIA3 does not have a direct impact on B. burgdorferi viability in the tick vector. Transmission of B. burgdorferi to the vertebrate host was also not affected when mice were fed upon by *ispdia3*-silenced B. burgdorferi-infected ticks. B. burgdorferi burden in ear skin distal from the tick bite sites was observed to increase from about 7 to 14 days post tick detachment, consistent with the kinetics of B. burgdorferi dissemination (28). While Hodzic et al. (28) showed that upon infection of mice by needle inoculation, B. burgdorferi burden in distal skin remained stable for 3 to 4 weeks, we observed a decrease around 3 weeks post tick detachment, potentially due to immune-mediated clearance of spirochetes. It is also likely that infection by tick bite impacts the dissemination kinetics.

An earlier study by Narasimhan et al. (22) found that silencing of *salp25D*, a secreted salivary peroxiredoxin, decreased B. burgdorferi colonization by altering the levels of reactive oxygen species at the vector-host interface. Although Narasimhan et al. (22) achieved preferential silencing of the gut or salivary gland Salp25D by anal pore (targeting the gut) or body (targeting the salivary gland) injections of specific dsRNAs to decipher the specific contribution of the gut or salivary Salp25D function, we could not achieve preferential silencing of gut or salivary gland IsPDIA3. Therefore, we could not attribute the observed phenotype of impaired B. burgdorferi colonization specifically to either the gut or salivary IsPDIA3.

Since RNAi-mediated silencing of *ispdiA3* in B. burgdorferi-infected ticks did not impact B. burgdorferi burden in the tick, we hypothesized that IsPDIA3 is likely to function at the vector-host interface to facilitate spirochete acquisition by the tick. Consistent with this notion, when we allowed *IsPDIA3* knockdown nymphs to feed on mouse ear skin, we observed increased expression of cytokines and chemokines, emphasizing the potential immunomodulatory role of IsPDIA3 at the skin-host interface. Cytokine/chemokine profiles of the bite sites of mice fed upon by IsPDIA3 knockdown ticks showed that IL-17, a cytokine expressed by Th17 cells was increased. IL-17 is a key cytokine that acts on epithelial keratinocytes to increase recruitment of inflammatory cells to the skin (29). Several other proinflammatory cytokines, including IL-4 and IL-1β, were also upregulated at bite sites when IsPDIA3 was knocked down in ticks. Chemokines such as CXCL2 and CXCL15 increased at the tick bites sites when IsPDIA3 was knocked down, which is expected to result in increased recruitment of macrophages and neutrophils.

We speculated that IsPDIA3 modulates inflammatory responses by binding to host immune effectors at the tick bite site. A functional screen for potential IsPDIA3 interactants using a human exoproteome yeast surface display library suggested that IsPDIA3 binds to insulin growth factor-like family receptor 1 (IGFLR1), also known as transmembrane protein 149 (TMEM149). This is a 355-amino-acid transmembrane protein that is primarily expressed on T cells (30). TMEM149 bears structural similarities to tumor necrosis factor (TNF) receptor family members, and human and mouse IGFLR1/TMEM149 share 61% amino acid sequence identity (30). Insulin growth factor-like protein (IGFL) was shown to be a ligand for IGFLR1 (30). IGFL is induced by TNF-α and was shown to be increased in skin in psoriasis and wounding models (29). The downstream signaling pathways initiated by the IGFL-IGFLR1 interaction is not fully understood. The physiological relevance of IsPDIA3-TMEM149 interaction in dampening inflammatory responses in the skin remains to be characterized in further detail.

It is interesting to note that TMEM149, like the TNF receptor family members, contains several cysteine-rich modules (31), and in several of these modules the cysteine residues have a conserved register that are known to form intrachain disulfide bridges critical for the structural and functional integrity of this family of proteins (31). This raises the possibility that the reductase activity of IsPDIA3 could alter these integral disulfide bonds that are critical for the function of TMEM149. Detailed studies to confirm the nature of interactions between IsPDIA3 and TMEM149 and the relevance of the enzymatic activity of IsPDIA3 in modulating TMEM149 will be critical to decipher the function of IsPDIA3 at the vector-host interface and to also expand our understanding of TMEM149 in T-cell biology.

We speculate that suppression of immune responses at the bite site facilitates B. burgdorferi survival and exit from the skin during colonization of the spirochete from the murine host by the tick. While such an advantage would also be expected during transmission from the tick to the mammalian host, this was not the case. This could be multifactorial, as the B. burgdorferi proteome is vastly different when it enters the tick versus when it exits the tick (32) and this could differentially impact the vulnerability of the spirochete at the tick-host interface. It is also likely that spirochetes exiting the tick are shielded by tick salivary proteins such as Salp15 (33). A detailed understanding of the specific effector molecules modulated by IsPDIA3-TMEM149 interaction would help clarify the differential impact of IsPDIA3 on colonization and transmission.

PDI homologs have been identified from other tick species, including Amblyomma variegatum and Haemaphysalis longicornis (19–21). H. longicornis PDIs have been invoked in blood feeding, cuticle formation, and oviposition (21) due to the broad functional role of PDIs in protein folding (34). The I. scapularis genome encodes at least four putative PDI paralogs. Analysis of the primary sequences of these paralogs showed that they shared 60 to 70% homology and that they all contain a canonical signal peptide, suggesting that they are all secreted proteins and may represent a family of proteins with related functions. Our observations suggest that IsPDIA3 modulates host immune responses at the vector-host interface during spirochete exit from the vertebrate host and entry into the tick gut. Since IsPDIA3 is expressed both in the salivary glands and in the gut, we cannot rule out the possibility that IsPDIA3 secreted in the gut might additionally enhance B. burgdorferi survival in the gut as it colonizes the gut epithelium. While the precise mechanism by which IsPDIA3 facilitates B. burgdorferi colonization remains to be deciphered, IsPDIA3 is an example of a tick protein that influences B. burgdorferi persistence in its infectious cycle, and targeting this protein may impair the ability of this spirochete to efficiently complete its life cycle.

## MATERIALS AND METHODS

### Ethics statement.

Animal care and housing followed the rules described in the Guide for the Care and Use of Laboratory Animals of the National Institutes of Health, USA. The protocols described below for the use of mice were reviewed and approved by the Yale University Institutional Animal Care and Use Committee (YUIACUC) and the approved protocol number is 2018-07941. All animal experiments were conducted in a biosafety level 2 animal facility according to YUIACUC rules.

### Cells and animals.

A low-passage-number B. burgdorferi N40 culture was grown without aeration in BSK-H medium (Sigma-Aldrich) at 32°C and frozen at −80°C in BSK-H supplemented with 20% glycerol. One Shot TOP10 chemically competent Escherichia coli (Thermo Fisher Scientific) and BL21(DE3) competent E. coli (Thermo Fisher Scientific) were used for cloning and protein expression, respectively, and were grown at 37°C with aeration in lysogeny broth supplemented with ampicillin (100 μg/ml). Expi293F was cultured in Expi293 expression medium (Thermo Scientific), and HEK-293T and Jurkat cell lines were maintained in Dulbecco’s modified Eagle medium (DMEM) supplemented with 10% fetal bovine serum (FBS; Gibco) and 1% penicillin/streptomycin (Gibco). C3H/HeJ mice were purchased from Jackson Laboratory. Infected mice were generated by subcutaneous injection of 100 μl of 1 × 10^5^ B. burgdorferi N40/ml. *Borrelia* burden was assayed by qPCR quantitation of spirochete DNA in mouse ear punch biopsy specimens collected 2 weeks after inoculation as described below.

### I. scapularis ticks.

I. scapularis larvae were acquired from the Centers for Disease Control and Prevention, and larvae were used to generate nymphs. Larval ticks were fed to repletion on pathogen-free C3H/HeJ mice and allowed to molt to nymphs. To infect nymphs, B. burgdorferi-infected mice were used. To limit the variability of ticks, all ticks (i.e., both control and test samples) within a single experiment were from the same egg clutch.

### RNAi silencing of *ispdiA3*.

**FIG 8:**
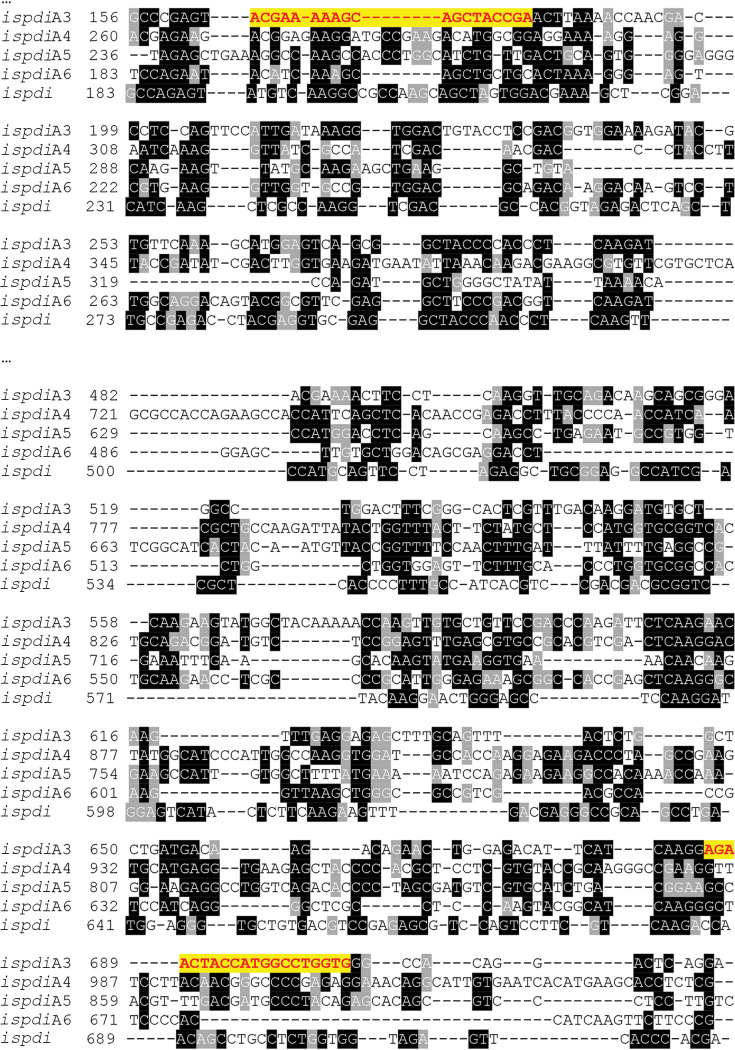
DNA sequence of I. scapularis PDIs. T-Coffee alignment of *ispdiA3* nucleotide sequence (ISCW016161) with other protein disulfide isomerases (PDIs) in I. scapularis. Nucleotide sequences shown in black are identical. The nucleic acid sequences in yellow show the sequences of dsRNA primers.

Fed-nymph salivary gland cDNA was prepared as described previously (35) and used as a template to amplify DNA encoding a fragment of *ispdiA3* (542 bp; ISCW016161). The PCR primers with T7 promoter sequences are shown in Table S1 in the supplemental material. The primer for dsRNA specifically amplifies *ispdiA3*, but not other IsPDI genes (Fig. 8). Complementary dsRNA was synthesized *in vitro* using the Megascript RNAi kit (Invitrogen, CA, USA). Clean or B. burgdorferi-infected I. scapularis nymphs were injected in the anal pore or idiosoma with ds *ispdiA3* or *ds gfp* control (6 nl, 3 × 10^12^ molecules) using glass capillary needles (36). To determine knockdown efficiency, ticks were fed on mice for 96 h, and samples were collected and analyzed as described below.

### Purification of recombinant IsPDIA3.

The coding region of *IsPDIA3* was PCR amplified from I. scapularis nymph cDNA using the primer pair listed in Table S1, then cloned into the BamHI and XhoI sites of the PGEX-6P-2 vector and the XbaI and NotI sites of the pEZT_D-lux vector. The PGEX-6P-2-IsPDIA3 plasmid produced an 81-kDa recombinant GST-IsPDIA3 (rIsPDIA3-GST) fusion protein in E. coli that was further purified on a glutathione Sepharose 4B column (GE, Pittsburgh, PA) as described by the manufacturer. The control GST fusion tag (rGST) was also purified using the empty PGEX-6P-2 vector. rIsPDIA3-GST, and rGST endotoxin were removed by high-capacity endotoxin removal resin (catalog no. 88273; Pierce) for intraperitoneal injection. *IsPDIA3* expression from the pEZT_D-lux plasmid produced a 60-kDa recombinant His-IsPDIA3 (rIsPDIA3-His) fusion protein in Expi293F cells and was purified on Ni-nitrilotriacetic acid (NTA) agarose (Qiagen, Valencia, CA) as described by the manufacturer.

### Western blot analysis of protein expression.

Recombinant proteins were placed in Laemmli sample buffer (Bio-Rad) and separated by SDS-PAGE using 4% to 20% Mini-Protean TGX gels (Bio-Rad). Proteins were transferred onto a 0.45-μm-pore-size polyvinylidene difluoride membrane and processed for immunoblotting. The immunoblots were incubated separately with rabbit polyclonal antibodies against rIsPDIA3-GST, anti-GST (Thermo Fisher Scientific, CA, USA) or anti-His monoclonal antibody (Thermo Fisher Scientific) at a dilution of 1:1,000 overnight at 4°C on a rocking platform. Bound antibodies were detected by using horseradish peroxidase-conjugated goat anti-rabbit or anti-mouse secondary antibodies (Invitrogen) at a dilution of 1:10,000 for 1 h at room temperature. The immunoblots were developed using the Amersham ECL Western blotting detection reagent (Sigma-Aldrich, St. Louis, MO), and the images were developed with a LI-COR Odyssey imaging system.

### B. burgdorferi colonization.

In experiments to address *Borrelia* colonization, at least 20 pathogen-free I. scapularis nymphs (ds *gfp* or ds *ispdiA3* injected into the body or anal pore as described above) were placed on each B. burgdorferi-infected mouse and allowed to feed to repletion. At least three mice were used in each experiment, and 10 to 12 ticks were collected from each mouse. Guts were dissected and analyzed for *Borrelia* colonization using qPCR. In experiments to address whether *Borrelia* colonization is affected by passive immunization, antibodies against rIsPDIA3-GST and rGST were produced in New Zealand White rabbits at Cocalico Biologicals, Inc. Whole serum (200 μl) was injected intraperitoneally 24 h prior to tick placement and at the time of larvae or nymph placement. For all colonization experiments, at least three mice were used in each group, and at least 25 nymphs and 60 larvae were analyzed per group.

### B. burgdorferi transmission.

To conduct *Borrelia* transmission studies, a group of three to five *gfp* or *ispdiA3* dsRNA-injected B. burgdorferi (N40)-infected nymphs were placed on each C3H/HeJ mouse (at least five mice each in the *gfp* or *ispdiA3* dsRNA groups) and allowed to feed for 66 h. Ticks were placed on the mouse head/back between the ears. Ticks were gently removed, and guts and salivary glands were dissected and processed in pools of three salivary glands and two guts for qPCR analysis as described below. At 7 and 14 days post tick detachment, the mice were anesthetized and skin samples were aseptically punch biopsied and assessed for spirochete burden by qPCR. Ticks fed in head area, and skin punch biopsy specimens were collected from the pinnae/ears. This site is considered distal, as it is not at the site of the tick bite. At 21 days post tick detachment, the mice were sacrificed, and ear skin, heart, and joints were aseptically collected and assessed for spirochete burden by qPCR.

### Tick RNA isolation, reverse transcription, and quantitative PCR (qRT-PCR).

The dsRNA microinjected nymphs were allowed to feed on naive or B. burgdorferi-infected C3H/HeJ mice. Salivary glands and guts were harvested in TRIzol, and total RNA was isolated according to the manufacturer’s protocol (Invitrogen). Fed whole larvae were pooled in groups of five, ground under liquid nitrogen, and resuspended in TRIzol to isolate RNA according to the manufacturer’s protocol (Invitrogen). cDNA was synthesized using the iScript cDNA synthesis kits (Bio-Rad, CA), and quantitative PCR was performed using iQ Syber Green Supermix (Bio-Rad) on a Bio-Rad cycler (Bio-Rad) with a program consisting of an initial denaturing step of 2 min at 95°C and 45 amplification cycles consisting of 20 s at 95°C, followed by 15 s at 60°C and 30 s at 72°C. The genes and corresponding primer sequences are shown in Table S1. For *flagellinB* (*flaB*), *ispdiA3*, and tick *actin*, specific target transcripts were quantified by extrapolation from a standard curve derived from a series of known DNA dilutions of each target gene, and data were normalized to tick *actin*.

### Mouse DNA isolation and quantitative PCR.

DNA was extracted from mouse skin punch biopsy specimens, joints, and hearts using a DNeasy tissue kit (Qiagen, Valencia, CA) according to the manufacturer’s protocol.

The resultant DNA was analyzed by qPCR as described above for the presence of Borrelia using *flaB* primers, and data were normalized to mouse β-*actin*. Primer sequences are shown in Table S1.

### Immunofluorescence microscopy.

Immunofluorescence was performed as previously described (37). Guts from nymphal ticks fed for ∼48 to 96 h were dissected, fixed in 4% paraformaldehyde (PFA) for 20 min, washed in phosphate-buffered saline (PBS)/0.5% Tween 20 (3 times), and blocked in PBS/0.5% Tween 20 and 5% fetal calf serum for 1 h prior to incubation with polyclonal rabbit anti-B. burgdorferi-N40 (1:1,000; Abcam, MA, USA) antibody, and bound antibodies were detected using Alexa Fluor 488-conjugated goat anti-rabbit antibody (1:500; Thermo Fisher Scientific) to visualize B. burgdorferi spirochetes.

### Immune responses at the tick bite site.

To determine the cytokine and chemokine profiles that may be modulated by IsPDIA3, clean nymphs microinjected with ds *ispdia3* or ds *gfp* were fed on the pinnae of clean mice. At 72 h post tick attachment, mice were sacrificed and ears dissected for RNA extraction and qPCR assessment of the expression profiles of different cytokines and chemokines. All of these cytokine and chemokine levels in skin tissues were normalized to mice β-*actin* RNA levels according to threshold cycle (2^−Δ^*^CT^*) calculations. Primers utilized for qPCR assessment are shown in Table S1.

### Yeast library screening.

Details of library construction and selections are described elsewhere (C. Rosen, unpublished data). Briefly, a library of barcoded plasmids containing the extracellular portions of 1,024 human proteins was expressed in Saccharomyces cerevisiae strain JAR300. Expression of surface protein display was induced by culturing the library in medium containing 90% galactose and 10% glucose for 24 h at 30°C. Induced yeast cells (10^7^) were pelleted in a sterile 96-well V-bottomed microtiter plate. Yeast cells were resuspended in 50 μl PBE (PBS plus 0.5% wt/vol bovine serum albumin [BSA] plus 0.5 mM EDTA) with 1 μl of protein (1 μg/μL rIsPDIA3-GST or rGST) added, then incubated for 1 h at 4°C with shaking. Yeast cells were washed once with 200 μl PBE, resuspended in 100 μl PBE with a 1:50 dilution of anti-GST antibody, and incubated for 1 h at 4°C with shaking. Yeast was washed once with 200 μl PBE and resuspended in 100 μl PBE with a 1:100 dilution of streptavidin microparticles (0.29 μm, catalog no. SVM-025-5H; Spherotech) and incubated for 1 h at 4°C with shaking. Yeast was washed once with 200 μl PBE, and bead-bound cells were selected by magnetic separation and subsequently expanded in 1 ml synthetic drop-out medium (SDO-Ura, catalog no. D9535, prepared according to the manufacturer’s instructions with 20 g/liter glucose; United States Biological) supplemented with chloramphenicol at 30°C. DNA was extracted from selected yeast cell libraries using Zymoprep-96 yeast cell plasmid miniprep kits or Zymoprep yeast cell plasmid miniprep II kits (Zymo Research) according to standard manufacturer protocols. DNA was amplified with custom primers and sequenced using an Illumina MiSeq instrument and Illumina v2 MiSeq reagent kits according to standard manufacturer protocols. Barcode counts were extracted from raw next-generation sequencing (NGS) data using Python. All enrichment calculations were performed using edgeR (38). The score for each gene is defined as the overall enrichment for that gene multiplied by the percentage of barcodes associated with that gene that are enriched (defined as a log of the fold change between sample and baseline [logFC] value of >0). Proteins with only 1 or 2 associated barcodes were excluded from analysis, leaving 766 analyzed proteins. The background corrected score is defined as the score for rIsPDIA3-GST minus the score for rGST alone.

### Bioinformatic analysis.

Multiple alignment of DNA and protein sequences was performed using T-Coffee (http://tcoffee.crg.cat/apps/tcoffee/do:regular) (39), and the alignment was formatted using BoxShade (http://www.ch.embnet.org/software/BOX_form.html). Protein homologs were identified using the BLASTP search tool (40). Signal sequence was determined using the SignalP 5.0 server (http://www.cbs.dtu.dk/services/SignalP/) (41).

### Statistical analysis.

Statistical significance of differences observed in experimental and control groups was analyzed using Prism version 8.0 (GraphPad Software, Inc., San Diego, CA). A nonparametric Mann-Whitney test was utilized to compare the mean values of control and test groups, and a *P* value of <0.05 was considered significant.

### Data availability.

All data generated in this work will be readily shared and are available upon request.

## Supplementary Material

Supplemental file 1
