# Supplementary material for: An Ixodes scapularis Protein Disulfide Isomerase Contributes to Borrelia burgdorferi Colonization of the Vector
Source: Infect Immun. 2020 Nov 16;88(12):e00426-20. doi: 10.1128/IAI.00426-20 (PMC7671890; doi:10.1128/IAI.00426-20)
Supplement: Supplemental file 1 [file IAI.00426-20-s0001.pdf]

Supplementary Table 1. Primers utilized in this study for quantitative RT-PCR assessment of gene expression profiles of *ispdiA3* gene clone, mice cytokines and chemokines

Gene Name	Forward primer sequence	Reverse primer sequence
Tick actin	*ggcgacgtagcag*	*ggtatcgtgctcgactc*
Mouse β-actin	*agcgggaaatcgtgcgtg*	*cagggtacatggtggtgcc*
Bb flaB	*ttcaatcaggtaacggcaca*	*gacgcrrgagaccctgaaag*
ds gfp	*taatacgactcactataggg gcgacgtaaacggccacaagtt*	*taatacgactcactatagggcg cgggtcttgtagttgccgtc*
ds ispdiA3 (ISCW016161)	*taatacgactcactataggg acgaaaaagcagctaccgaa*	*taatacgactcactataggg caccaggccatggtagttct*
ispdiA3_pEZT_Dlux	*ctag tctaga agcgatgtcctcgactactcggcggacttcgaca*	*ttttcctttt gcggccgc ctcctccttggccctcttggctc*
ispdiA3_PGEX-6P	*ggatcc gacatggtg agcgatgtcctcgactactcggcgg*	*ctcgag taactcctccttggccctcttggctcc*
ispdiA3 qPCR	*ttgccagcgatgtcctcgac*	*ctcttgcagtgtccgcacca*
CXCL15(IL-8)	aacctaggcatcttcgtccg	ttcacccatggagcatcagg
IP-10/CXCL10	gtctgagtgggactcaagggat	aggctcgcagggatgatttc
IL-1β	gcagtggttcgaggcctaat	gctgcttcagacacttgcac
CXCL2	cccagacagaagtcatagccac	cttccgttgagggacagcag
IL-18	gactcttgcgtcaacttcaagg	caggctgtcttttgtcaacga
TNF-α	aggcactcccccaaaagatg	tggtggtttgtgagtgtgagg
IFN-γ	gaggaactggcaaaaggatgg	acctgtgggttgttgacctc
MIP-1α/CCL3	gccaggtgtcattttcctgac	ctcaagcccctgctctacac
MIP-1β//CCL4	agaagacaccagagcatcacc	gagggtcagagcccattggt
RANTES/CCL5	gacagcacatgcatctccca	gtgtccgagccatatggtga
IL-6	ataccactcccaacagacct	ccagtttggtagcatccatc

IL-23	tggctgtgcctaggaagtagca	ttcatcctcttcttctcttagtagattcata
IL-10	gtacagccgggaagacaataac	gcattaaggagtcggttagcag
IL-4	cggatgcgacaaaaatcac	cgtttggcacatccatctc
IL-5	atggagattcccatgagcac	tctccaatgcatagctggtg
IL-12	atcgttttgctggtgtctcc	cttcttcaggcgtgtcacag
TGF-β	tggagcaacatgtggaactc	tgccgtacaactccagtgac
IL-17	tcatctgtgtctctgatgctgttg	tcgctgccttcactgt
CXCR1	aatctgttgtggcttcaccca	gctatcttccgccaggcatat
CXCR2	gaaatttcgccatggacttctc	acgagctaacaaaagaaggcctt
CXCL10	gccgtcattttctgcctcat	gcttccctatggccctcatt
KC	tcgccaatgagctgcgctgtc	gcttcagggtcaaggcaagcc
VACM	cttgggagcctcaacggtact	gcccgtagtgctgcaagtg
CXCL4	cagtcctgagctgctgcttct	tccaggctggtgatgtgctta
ICAM-1	tgcgttttggagctagcggacca	cgaggaccatacagcacgtgcag
IL-2	aagctctacagcggaagcac	atcctggggagtttcaggtt
FOXP3	cccacacctcttcttccttg	gtggtctgtcctggagaagg
GCP2	tggatccagaagctcctgtga	tgcattccgcttagcttcttt
CCL2	ttaaaaacctggatcggaaccaa	gcattagcttcagatttacgggt
CCL20	cttgctttggcatgggtact	tgtacgagaggcaacagtcg
TLR2	aagaggaagcccaagaaagc	aatgggaatcctgctcactg
